# Hepatorenal syndrome:Response to terlipressin and albumin and its determinants

**DOI:** 10.12669/pjms.322.9315

**Published:** 2016

**Authors:** Shahid Sarwar, Anwaar A. Khan

**Affiliations:** 1 Dr. Shahid Sarwar, FCPS (Medicine) FCPS (Gastroenterology). Associate Professor Gujranwala Medical College, Consultant Gastroenterologist, Doctors Hospital & Medical Center, Lahore, Pakistan; 2 Anwaar A. Khan, MACP, FACG, FRCP, AGAF, FCPSEx- Dean and Professor of Gastroenterology, ShaikhZayed Post Graduate Medical Institute, Consultant Gastroenterologist, Doctors Hospital & Medical Center, Lahore, Pakistan

**Keywords:** Cirrhosis, Hepatorenal syndrome (HRS), intravenous albumin, Terlipressin

## Abstract

**Objective:**

To determine the efficacy of terlipressin and albumin in improving renal functions in patient with hepatorenal syndrome (HRS) and to identify factors determinant of better response.

**Methods:**

In this quasi experimental interventional study patients of liver cirrhosis and ascites with HRS type I were treated with intravenous albumin and incremental dosage of terlipressin based on response with maximum dose of 12mg/day. Decline of creatinine below 1.5mg/dl was defined as complete response. Factors predictive of response to therapy were determined via linear regression analysis.

**Results:**

Twenty four patients were included with male to female ratio 3.8/1(19/5) and mean age 53.3 (±10.06). Complete response to terlipressin/albumin was seen in 14 (58.3%)patients, seven (29.2%) achieved partial response with > 25% creatinine decline while three (12.5%) had no response. Lower serum creatinine at diagnosis (P value 0.003), absence of hyperkalemia (p value 0.005) and absence of portal vein thrombosis (p value 0.05) are associated with response to treatment in HRS. Baseline serum creatinine (p value 0.003) was independent predictor of response to therapy in multivariate analysis.

**Conclusion:**

Terlipressin and albumin is an effective treatment for HRS type I. Patients with lower baseline serum creatinine are more likely to respond to this therapy.

## INTRODUCTION

Hepatorenal syndrome is a fatal but potentially reversible complication of end stage liver disease. It is defined as impairment of renal function in a patient with liver cirrhosis in the absence of any alternative identifiable cause of renal failure.^1^ Due to absence of biochemical or radiological diagnostic indicators, diagnosis of HRS is primarily of exclusion. Deranged renal profile in a patient with cirrhosis and ascites in the absence of alternative causes of renal impairment and failure to respond to trial of intravenous albumin is sufficient to diagnose HRS.^2^ It is classified as type 1 and 2 based on value of serum creatinine and time it has taken to worsen.^3^ Median survival for patients with HRS is three months. Outcome is especially dismal in type 1 HRS where survival without treatment is one month.^4^

What triggers HRS remained the source of controversy over many years. Now we have consensus that splanchnic arterial vasodilatation resulting from excess of vasodilators in circulation in a patient with cirrhosis is the primary trigger for HRS.^5^ Many authorities now believe that this vasodilatation is a consequence of systemic spread of bacterial products following induction of inflammation by host micorbiota, resulting in endothelial injury more pronounced in splanchnic circulation.^6^ Splanchnic vasodilatation results in reduced effective circulatory volume with renal hypo-perfusion which is further augmented by renal arterial vasoconstriction due to sympathetic over-activity and excess angiotensinogen II levels.^7^ HRS is a functional renal disorder which can be reversed by either expanding plasma volume or by inducing splanchnic vasoconstriction thus improving renal perfusion.

Combination of splanchnic vasoconstrictor along with intravenous albumin for volume expansion is treatment of choice for HRS as per Acute Dialysis Quality Initiative (ADQI) work group recommendations.^8^ It results in improved renal perfusion with normalization of sympathetic over-activity as well as of angiotensinogen II levels. Drug which has shown best results in combination with albumin is terlipressin, which is a vasopressin analogue that acts on vasopressin receptors and leads to splanchnic vasoconstriction. Several meta-analyses have shown 40-50% response rate in patients with type I HRS with this treatment.^9^,^10^ However a multicenter randomized controlled trial in 56 patients with HRS comparing terlipressin to placebo found similar survival for both groups at 180 days (42.9% versus 37.5%, P = 0.8). Other treatment options include noradrenaline, midodrine in combination with octreotide, trans jugular intrahepatic Porto systemic shunt (TIPS) and extracorporeal support systems. None of these have convincing evidence of improved outcome.^11^

Inasmuch as we have effective therapeutic options for variceal bleeding, ascites or encephalopathy, we are encountering increasing number of patients with HRS. Identification of cost effective treatment for HRS in our patients with cirrhosis is urgently needed. Moreover, in view of potential side effects related to vasoactive drug, we would like to limit this treatment to patients most likely to respond by identifying factors associated with better outcome. Objective of our study was to determine the effectiveness of terlipressin in combination with albumin in patients with HRS Type-1 in improving renal functions and to identify factors associated with favorable outcome of this treatment.

## METHODS

This experimental design cohort study was carried out at The Doctors Hopsital & Medical Center from January 2008 to June 2015. Only patients with Type-1 HRS were included. Sampling technique was non-probability purposive convenient sampling. Patients with cirrhosis and ascites, as confirmed on abdominal ultrasound who had doubling of creatinine above 2.5mg/dl within two weeks were included. HRS was confirmed by absence of shock at admission, absence of hypovolemia as confirmed by “failure to improve renal function (decrease in creatinine < 1.5 mg/dl) following atleast 2 days of diuretic withdrawl (if on diuretics), and volume expansion with albumin at 1 g/kg/day up to a maximum of 100g/day”, no current or recent treatment with nephrotoxic drugs, absence of parenchymal renal disease as defined by proteinuria < 0.5 g/day, no haematuria (< 50 red cells/high power field) and normal renal ultrasound.^3^ Patients with history of ischemic heart disease were excluded.

After detailed clinical history and examination, complete blood count, liver function tests, renal function tests, serum electrolytes, urine complete examination and abdominal ultrasound were carried out in all patients. All patients were given intravenous albumin 1g/kg up to maximum of 100 gm to exclude hypovolemia before confirmation of HRS. Ascitic fluid was examined for differential count, biochemistry and culture.

Patients were treated with terlipressin, initially 2mg/day along with intravenous albumin 20mg/day. Response was evaluated through monitoring of vital signs, daily urine output, daily serum creatinine and clinical condition of patient. If serum creatinine failed to decline by 25% of baseline value after three days, dose was increased to 4mg, 8mg and 12mg/day progressively. Maximum dose limit was 12mg/day. Dose of terlipressin was not increased further in case of favorable response defined as ≥ 25% reduction in creatinine within three days time or maximum dose limit reached. Complete response to terlipressin in combination with albumin was defined as decline in serum creatinine < 1.5 mg/dl. Decline of more than 25% of baseline creatinine but not below 1.5 mg/dl in creatinine value was regarded as partial response. Less than 25% decline in baseline creatinine with maximum dose possible was defined as No response.

Patients with variceal bleeding, spontaneous bacterial peritonitis or portosystemic encephalopathy were treated as per standard protocol. Patients with complete or partial response and improvement in their clinical condition were advised medications and evaluation by liver transplantation unit at discharge. In case of no response, due to absence of alternative treatment options urgent liver transplantation was recommended.

### Statistical analysis

Statistical analysis was carried out using SPSS^®^ 20. Quantitative variables were expressed as mean ± standard deviation (SD) while qualitative variables were given as percentage. Complete, partial or no response were given as percentage. Univariate analysis to identify variables associated with response to treatment were determined using chi squareχ^2^ for qualitative and unpaired two tailed student’s t test for quantitative variables. Variables with p value ≤0.1 were used for multivariate analysis for independent prediction of response to therapy by multivariate linear regression analysis. Reciever operating characteristic (ROC) curve was used to identify cut off value of variables predictive of response to therapy.

## RESULTS

Total of 24 patients diagnosed with HRS Type-I were included. Male to female ratio was 3.8/1(19/5) with mean age of 53.3 (±10.06) years. Hepatitis C was responsible for liver cirrhosis in 21(87.5%) patients, one (4.2%) patient had hepatitis B, whereas two were negative for both hepatitis B and C. Hepatocellular carcinoma was already diagnosed in 10 (41.75%) patients. On clinical evaluation all patients had ascites and jaundice, 13 (54.2%) presented with abdominal pain, 9 (37.5%) with fever, 23 (95.8%) complained of oliguria, 22 (91.7%) had portosystemic encephalopathy, four (16.7%) presented with upper gastrointestinal bleeding and three (12.5%) complained of dyspnea. Ascites was mild to moderate in 16 (66.6%) patients while 8 (33.4%) had tense ascites. Spontaneous bacterial peritonitis was confirmed on ascitic fluid analysis in seven (29.1%) patients. All patients were in Child Pugh Turcotte (CTP) class C with mean score of 13.46 (±1.1) whereas mean Model for End stage Liver Disease (MELD) score was 36.6 (±5.5). Only one (4.2%) patient had MELD score less than 30, 16(66.7%) had score between 30-39 while 7(29.2%) had score of 40. Hyponatremia was present in 16 (66.7%) patients while 7 (29.2%) had hyperkalemia.

Maximum dose of terlipressin used was 2mg/day in 5 (20.8%), 4mg/day in 16(66.7%) patients and 8mg/day in 3 (12.5%) patients.

Complete response of HRS to terlipressin/albumin was seen in 14 (58.3%) patients, seven (29.2%) achieved partial response while three (12.5%) had no improvement in renal profile. Mean time to achieving serum creatinine <1.5 mg/dl in patients with complete response was 5.14 (±1.14) days. Of patients with complete response, 13 (92.8%) were discharged and referred for liver transplantation while 1 (7.2%) died due to worsening encephalopathy. Among patients with partial response, maximum dose limit of terlipressin was not reached as 5 (71.4%) died during treatment, one due to hyperkalemia induced arrythmia and 4 due to multi-organ failure whereas two (28.6%) were shifted to transplant center during treatment. All three patients with no response died during hospital admission. Referral for transplantation was possible in 15 (62.5%) patients while 9 (37.5%) died during treatment.

**Fig.1:**
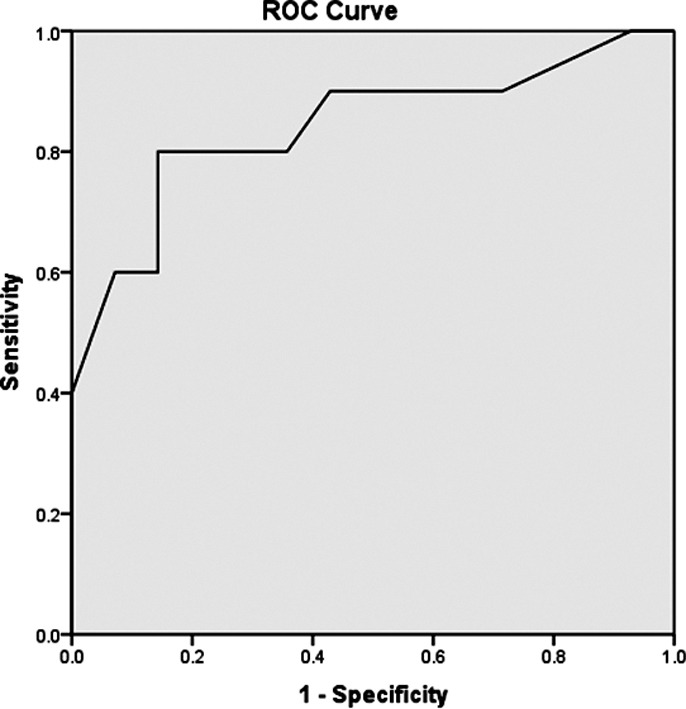
Correlation of high baseline serum creatinine with failure to improve HRS. Area under curve: 0.853

We compared variables noted during study between patients with complete response and those who failed to achieve complete response to terlipressin/albumin, to identify predictors of successful treatment of HRS as given in Table-I. Age of patient (p value 0.09), lower serum creatinine at diagnosis (P value 0.003), absence of hyperkalemia (p value 0.005) and absence of portal vein thrombosis (p value 0.05) were associated with response to treatment in HRS. Baseline serum creatinine (p value 0.003) was sole independent predictor of response to therapy in multivariate analysis as shown in Table-II. Serum creatinine ≥ 3.5 mg/dl at baseline was associated with higher probability of failure to respond to terlipressin/albumin therapy as determined by ROC curve. Fig.1. No significant correlation was found between response to therapy in HRS and dose or duration of terlipressin or albumin.

**Table-I:** Comparison of patients with and those without response to treatment in HRS.

Variables	HRS settled (Mean±SD)	HRS not settled (Mean±SD)	P value
Age (years)	50.43(9.7)	57.5(9.5)	0.09
Duration of liver disease (month)	24.7(33.5)	32.2(41.2)	0.62
Hemoglobin (g/dl)	10.5 (2.2)	10.2 (2.02)	0.79
Platelet (x 109/L)	95.2 (39)	106.4 (69.5)	0.62
Prothrombin time (sec)	30.6 (9.1)	24.8 (4.8)	0.12
Bilirubin (mg/dl)	19.38 (13.2)	12.1 (9.7)	0.15
Albumin (g/dl)	2.4 (0.65)	2.47(0.6)	0.81
Creatinine (mg/dl)	3.2 (0.51)	4.6 (1.4)	0.003
Serum sodium (mEq/dl)	126.2 (7.5)	133.7 (6.4)	0.11
MELD score	37.7 (6.2)	35.1 (4.2)	0.26
CTP score	13.5 (0.76)	13.4 (1.5)	0.83
Hyperkalemia (No of patients)	1	6	0.005
Size of HCC lesion (cm)	1.2 (2.2)	3.07 (3.2)	0.12
Portal vein thrombosis (No of patients) (Total number-5)	1	4	0.05

*SD: Standard Deviation.

**Table-II:** Multivariate analysis for prediction of response to therapy.

Coefficients^a^							

	Model	Unstandardized Coefficients		Standardized Coefficients	t	Sig.

		B	Std. Error	Beta		
1	(Constant)	.482	.288		1.672	.109
	Serum creatinine	.245	.072	.586	3.393	.003

a, Dependent Variable: HRS improved or not

Model		Excluded Variables^a^			Partial Correlation	Collinearity Statistics
	
		Beta In	t	Sig.		Tolerance
	Age of patient	.145^b^	.762	.454	.164	.844
	Hyperkalemia during treatment	-.361^b^	-1.845	.079	-.374	.703
	Portal vein thrombosis	-.245^b^	-1.384	.181	-.289	.910

a Dependent Variable: HRS improved or not

b Predictors in the Model: (Constant), Serum creatinine

## DISCUSSION

Hepatorenal syndrome is one of the dreadful terminal complications encountered in patients with advanced cirrhosis. Failure to treat HRS especially type 1 is invariably fatal.^3^ All patients in our study had advanced cirrhosis with mean CTP score of 13.46. More than 40% patients had hepatocellular carcinoma in our patient population.

Complete response to combination of terlipressin and albumin was seen in 58.3% of patient in our study. In a study of 119 patients with HRS by Heidemann J et al., response rate was 55%.^12^ Nazar A et al. has shown 46% response with decline in creatinine below 1.5mg/dl in a study of 39 patients.^13^ Contrarily a small study by Licata A has shown dismal outcome with terlipressin and albumin with response rate of 9.1% in a study of 33 patients.^14^ Multiple meta-analytical studies reviewing randomized trials have noted cumulative response rate of varying from 40 to 60% as noted in our study.^15^,^16^

Important query to be answered is “Does this benefit translate in survival improvement”? Gluud LL et al. reviewed 5 randomized trials available on vasoconstrictor/albumin therapy in HRS and cumulative data showed reduced mortality with treatment (RR 0.76 95% CI 0.61-0.95).^17^ In another review by Fabrizi F et al. of 5 randomized studies with total of 243 patients, significant reversal of HRS with OR 8.09 95% CI(3.52-18.59) (p value 0.001 was noted but no significant effect on survival was seen with OR 2.06.^18^ Better survival was noted for those with response to terlipressin and albumin as compared to those with no response in a study of 119 patients by Heidemann J et al.^12^ Issue of survival benefit with vasoconstrictors in HRS is still far from settled but this treatment does buy time for arranging liver transplantation. Moreover outcome of transplantation is better for patients with normal renal functions prior to transplant surgery.^19^

High baseline serum creatinine, hyperkalemia, and portal vein thrombosis on ultrasound were identified as bad prognostic indicators in univariate analysis of our patients but only baseline creatinine stood the test of multivariate analysis as independent predictor of response to treatment with terlipressin in combination with albumin. Nazar A noted that bilirubin ≥ 10mg/dl and rise in mean arterial pressure (MAP) of ≥ 5mm are associated with poor response to therapy and lower baseline creatinine results in faster recovery.^13^ Boyer TD et al. concluded that serum creatinine < 3 mg/dl (0.029) and rise in MAP are associated with better response to treatment in HRS type I.^20^ Serum creatinine was the only predictor of response to terlipressin/albumin in a study by Martin-Llahi M et al.^21^ Sharma P et al. noted that patients with serum creatinine > 7 mg/dl are less likely to respond to vasoconstrictor therapy.^22^ Patients with serum creatinine ≥ 3.5 mg/dl were more likely to be non-responders of this treatment in our study.

Our study is limited by the absence of control group but as terlipressin is now recommended along with albumin for HRS Type- I, control group was not possible. Studies with larger number of patients comparing terlipressin with other vasoconstrictor agents may further enrich our knowledge regarding factors associated with better response. Identification of these factors will enable us to limit this costly treatment to patients most likely to respond thus saving cost of treatment. Very few studies pertaining treatment of HRS are available in literature from our region. As more and more centers are developing liver transplantation services in our country, studies like ours will enable us to select better treatment option to bridge patients of HRS to liver transplantation.

## CONCLUSION

Terlipressin along with albumin is an effective therapeutic intervention for HRS type I. Patients with lower baseline serum creatinine are more likely to respond to this therapy.
